# Polyphenolic Composition of *Carlina acaulis* L. Extract and Cytotoxic Potential against Colorectal Adenocarcinoma and Cervical Cancer Cells

**DOI:** 10.3390/molecules28166148

**Published:** 2023-08-20

**Authors:** Ireneusz Sowa, Jarosław Mołdoch, Roman Paduch, Maciej Strzemski, Jacek Szkutnik, Katarzyna Tyszczuk-Rotko, Sławomir Dresler, Dariusz Szczepanek, Magdalena Wójciak

**Affiliations:** 1 Department of Analytical Chemistry, Medical University of Lublin, Chodźki 4a, 20-093 Lublin, Poland; maciej.strzemski@poczta.onet.pl (M.S.); dresler.slawomir@gmail.com (S.D.); 2 Department of Biochemistry and Crop Quality, Institute of Soil Science and Plant Cultivation, State Research Institute, 24-100 Puławy, Poland; jmoldoch@iung.pulawy.pl; 3 Department of Virology and Immunology, Institute of Biological Sciences, Faculty of Biology and Biotechnology, Maria Curie-Skłodowska University, 19 Akademicka Street, 20-033 Lublin, Poland; roman.paduch@mail.umcs.pl; 4 Independent Unit of Functional Masticatory Disorders, Medical University of Lublin, 20-093 Lublin, Poland; jacek.szkutnik@umlub.pl; 5 Institute of Chemical Sciences, Faculty of Chemistry, Maria Curie-Skłodowska University in Lublin, 20-031 Lublin, Poland; katarzyna.tyszczuk-rotko@mail.umcs.pl; 6 Chair and Department of Neurosurgery and Paediatric Neurosurgery, Medical University of Lublin, 20-090 Lublin, Poland; dariusz.szczepanek@umlub.pl

**Keywords:** anticancer, traumatic acid, pinellic acid, stemless carline thistle, polyphenols

## Abstract

*Carlina acaulis* is highly valued in the traditional medicine of many European countries for its diuretic, cholagogue, anthelmintic, laxative, and emetic properties. Moreover, practitioners of natural medicine indicate that it has anti-cancer potential. However, its phytochemistry is still little known. In the present study, the polyphenolic composition of the plant was investigated using ultra-high-performance liquid chromatography coupled with a high-resolution/quadrupole time-of-flight mass spectrometer (UHPLC-HR/QTOF/MS-PDA). The fractionation of the extract was carried out using liquid-liquid extraction and preparative chromatography techniques. Cytotoxicity was assessed based on neutral red and MTT assays. The obtained data showed that the species is rich in chlorogenic acids and C-glycosides of luteolin and apigenin. The total amount of chlorogenic acids was 12.6 mg/g. Among flavonoids, kaempferol dihexosidipentose and schaftoside were the most abundant, reaching approximately 3 mg/g, followed by isoorientin, vitexin-2-O-rhamnoside, and vicenin II, each with a content of approximately 2 mg/g. Furthermore, the cytotoxic potential of the plant against human colorectal adenocarcinoma (HT29) and human cervical cancer (HeLa) cells was investigated using the normal epithelial colon cell line (CCD 841CoTr) as a reference. It has been demonstrated that the ethyl acetate fraction was the most abundant in polyphenolic compounds and had the most promising anticancer activity. Further fractionation allowed for the obtaining of some subfractions that differed in phytochemical composition. The subfractions containing polyphenolic acids and flavonoids were characterized by low cytotoxicity against cancer and normal cell lines. Meanwhile, the subfraction with fatty acids was active and decreased the viability of HeLa and HT29 with minimal negative effects on CCD 841CoTr. The effect was probably linked to traumatic acid, which was present in the fraction at a concentration of 147 mg/g of dried weight. The research demonstrated the significant potential of *C. acaulis* as a plant with promising attributes, thus justifying further exploration of its biological activity.

## 1. Introduction

**Figure 1:**
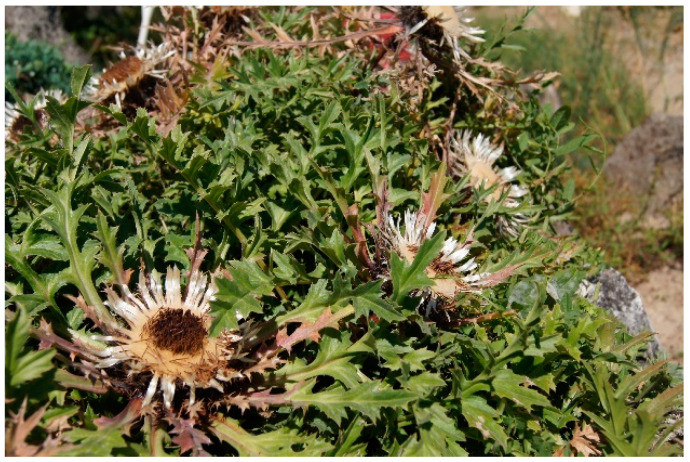
An example of *C. aculis* growing in its natural habitat.

*Carlina acaulis* is a perennial species belonging to the Carlina genus in the Asteraceae family. The plant mainly occurs in mountainous areas in South and Central Europe, in many countries including Poland, Germany, Italy, France, Austria, and Romania. The species is found in nature in xerothermic and calcareous grasslands, rocky regions, and dry meadows. A characteristic feature of the plant is its rosette (up to 20 cm in diameter), which is composed of elliptical-oblong, spiky, and pinnatilobate leaves and blossoms with silvery-white ray flowers that are grouped around a yellow-brown central disc [1,2]. The example of *C. aculis* plants found at the natural site is shown in Figure 1.

*C. acaulis* has been widely used in folk medicine in many countries. Extracts from the above-ground parts of the plant were applied as diuretics, cholagogues, anthelmintics, laxatives, and emetic agents. Furthermore, they were also used to stimulate the mental system, treat impotence, and alleviate skin disorders such as eczema and mycoses. The roots were mostly used externally for acne, eczema, and skin ulcers and internally as a diuretic, anthelmintic, and diaphoretic agent [3,4,5,6]. The inflorescences and roots are edible and can be used as food additives or to prepare drinks and snacks [1].

Nowadays, *C. acaulis* is the subject of interest for many researchers and intensive scientific investigations. The majority of reports are focused on the activity of root essential oil (EO) and carlina oxide, the predominant constituent of the EO, which has shown significant bactericidal, antifungal, antiviral, insecticidal, and acaricidal potential [7,8,9,10,11,12]. Furthermore, it has been evidenced that extracts from herbs and roots possess antioxidant, anti-inflammatory, anti-ulcer, and antimicrobial activities [13] and show a cytotoxic effect against human melanoma [14]. A phytochemical investigation of the species has revealed that the essential oil consists of 80% to even up to 99% of carlina oxide, followed by benzaldehyde, ar-curcumene, (E,Z)-α-farnesene, β-sesquiphellandrene, and 1,8-cineole [15,16]. In addition, *C. acaulis* is a rich source of triterpenic compounds [17] and phenolic acids, including chlorogenic, feruloylquinic, coumaroylquinic, and dicaffeoylquinic acids [18]. There are also mentions of the presence of flavonoids, mostly luteolin and apigenin derivatives [13,19]. Roots contain a high amount (approximately 20%) of inulin, a valuable polysaccharide with prebiotic properties.

As it can be seen, the state of knowledge on *C. acaulis* is still insufficient; therefore, the main goal of the paper was to expand the understanding of *C. acaulis*, particularly in the context of the qualitative and quantitative composition of polar components. The phytochemical analysis was carried out using modern chromatographic techniques, including LC-HRMS-QTOF-CAD and UHPLC-PDA-MS. Furthermore, the antiproliferative activity of the extract against human colorectal adenocarcinoma and human cervical cancer cells was investigated because, despite the usage of *C. acaulis* by traditional medicine practitioners as an anti-cancer drug [2], the number of experimental studies on this topic is very limited. Antioxidant effects were also included in the study, as antioxidants have great significance in cancer prevention.

## 2. Results

### 2.1. UHPLC-DAD-MS Analysis

**Figure 2:**
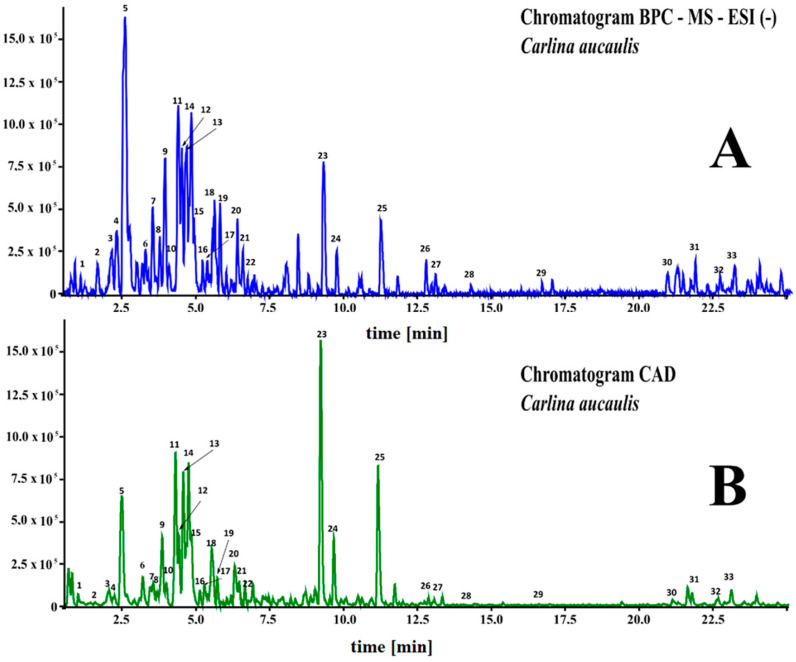
Chromatograms of extracts from *C. acaulis* obtained using a quadrupole-time-of-flight high-resolution mass spectrometer (LC-HRMS-QTOF) and a charged aerosol detector (CAD). (**A**) MS chromatogram (electrospray ionization—ESI); (**B**) CAD chromatogram. Numbering of the compounds as shown in Table 1.

Methanolic/water extracts from the above-ground parts of *C. acaulis* were separated, and their chemical composition was analyzed using UHPLC-MS. The compounds were identified based on mass data (m/z-H), fragmentation patterns, UV-Vis spectra, and additionally by comparison with standards when available. An example of the obtained chromatograms is shown in Figure 2.

A total of twenty different phenolic compounds were identified in the *C. acaulis* extract, including phenolic acids and flavonoids. Among the acids, 5-caffeoylquinic and 3-caffeoylquinic acids were the most abundant, with contents reaching up to 9.21 and 3.49 mg/g of dry weight, respectively. This was followed by dihydroxybenzoic acid (2.14 mg/g), quinic acid (0.99 mg/g), and 5-p-coumaroylquinic acid (0.57 mg/g).

From the flavonoid class, kaempferol dihexoside pentose (3.14 mg/g) was predominant, followed by C-glycosides of apigenin, namely shaftoside (6-C-glucoside-8-C-arabinoside), present at the highest amount, reaching 3.12 mg/g, isoschaftoside (6-C-arabinoside-8-C-glucoside) with a total content of 2.28 mg/g, vitexin-2-O-rhamnoside (1.96 mg/g), and isovitexin 2″-O-rhamnoside (1.74 mg/g). In addition, two derivatives of isovitexin, vicenin II and isovicenin, were found to have a content of 1.92 and 1.0 mg/g, respectively. The other flavonoids found in the aboveground part of C. acaulis were C-glycosides of luteolin, represented by isoorientin (6-C-glucoside), orientin (8-C-glucoside), 6-C-xylosyl luteolin, luteolin di-C-glucoside, and 2″-isoorientin O-glucopyranoside, with mean contents of 1.84, 1.05, 0.97, 0.69, and 0.41 mg/g, respectively. A small quantity of gossypetin dihexose (0.57 mg/g) and hesperitin 7-O-diglucosorhamnoside (0.2 mg/g) were also detected. Furthermore, several lipophilic constituents, including phospholipids 9,10-dihydroxy-8-oxooctadec-12-enoic acid, traumatic acid, and pinellic acid, are visible on the chromatogram of the methanol/water extract of *C. acaulis*.

The results of qualitative analysis and quantification, expressed per gram of dried plant material, are shown in Table 1.

### 2.2. Plant Material and Fractionation

Two years field cultivation of *C. acaulis* yielded 6.47 kg of plant material. After freeze-drying, the total weight of the lyophilized powder was 1.1 kg. Exhaustive extraction of 1 kg of freeze-dried samples with 70% methanol yielded 202 g of the first fraction (ECA).

ECA was subsequently fractionated using solvents with different polarities, including hexane, followed by ethyl acetate, and n-butanol, which yielded 5.62 g (HCA), 2.52 g (EaCA), and 5.26 g (BCA) of dried extract, respectively. An amount of 186 g of solid residues remaining after organic solvent extraction were dissolved in water (H_2_OCA).

#### 2.2.1. Chromatographic Analysis

The qualitative and quantitative phytochemical composition of the extracts was characterized, and the results were expressed per g of dried extract (Table 2). Examples of the UHPLC-MS and PDA chromatograms obtained for specific fractions are shown in Figure S1 (Supplementary Material).

Chromatographic analysis revealed that the ethyl acetate fraction was predominant in phenolic compounds, which constitute approximately 55% of the dry weight of the extract. The butanol and water fractions contained significantly lower amounts of polyphenols, approximately 11% and 1.1% of the dry weight, respectively. Phospholipids remained in the hexane fraction.

#### 2.2.2. Antioxidant Activity

The content of polyphenols in EaCA significantly affects free radical scavenging activity (DPPH) and ferric reducing power (FRAP) (Table 3). EaCA was the most potent free radical scavenger and had the highest ability to reduce ferric ions. BCA and H_2_OCA exhibited an almost equal effect, and HCV had the lowest antioxidant potential.

#### 2.2.3. Cytotoxic Activity

**Figure 3:**
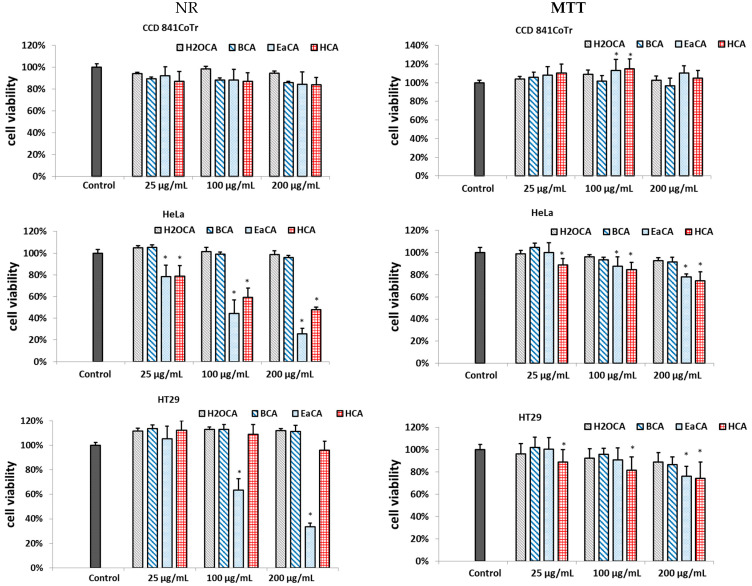
Effect of the different concentrations of fractions obtained from methanol/water extract of *Carlina acaulis* on cell viability as determined by the NR and MTT assays and expressed as a % of control (0.5% of DMSO in medium). The fractions were obtained using hexane (HCA), followed by ethyl acetate (EaCA), butanol (BCA), and water (H2OCA). The data are means (*n* = 3) ± SD. One-way ANOVA followed by Dunnett’s post hoc test; the differences were considered significant at *p* < 0.05. * indicates statistically significant difference.

In the study, two complementary tests were used to evaluate the cytotoxicity of the analyzed extracts against human colorectal adenocarcinoma (HT29) and human cervical cancer cells (HeLa), including MTT and the neutral red (NR) uptake assay. The MTT assesses cell metabolic activity based on the measurement of NAD(P)H-dependent cellular oxidoreductase. In turn, NR allows the detection of viable cells because the dye is retained in lysosomes in living cells. A normal epithelial colon cell line (CCD 841CoTr) was used as a reference line. The results are shown in Figure 3. The data indicated that all extracts at the tested concentration range were not toxic or showed mild toxicity against the reference line. In turn, EaCA and HCA exhibited cytotoxicity against HeLa, and EaCA also effectively reduced the number of viable cells in the HT29 line. For example, at a concentration of 200 µg/mL, it decreased the number of visible cells to 33% and 26% in HT29 and HeLa, respectively, while in CCD 841CoTr, viability stayed at the level of 84% (NR test). No effects were noted for BCa or H_2_OCA.

### 2.3. Bioactivity-Guided Subfractionation

#### 2.3.1. Chromatographic Analysis

The EaCA fraction, being the most active against the tested cancer lines, was chosen for further fractionation using 20% methanol (EaCA_1), followed by 60% methanol (EaCA_2), 80% methanol (EaCA_3), and 100% methanol (EaCA_4). Phytochemical analysis (Table 4) revealed that phenolic acids were accumulated in the first fraction, constituting approximately 43% of the total yield. In contrast, the second fraction was abundant in flavonoid compounds (approximately 68% of the total yield). EaCA_3 contained fatty acids, including traumatic, pinellic, and 9,10-dihydroxy-8-oxsooctadec-12-enic acid (40.1%), and no polyphenolic constituents were found in the fraction. In EaCA_4, no components were identified because they were not detected by MS (they were not ionizable) or UV-Vis detectors (they had no chromophores).

#### 2.3.2. Antioxidant Activity

As expected, EaCA_1 and EaCA_2 had the highest antioxidant activity because polyphenolic compounds were exhaustively extracted in the first two stages of the process. On the other hand, EaCA_3 and EaCA_4 had minimal free radical scavenging capacity and the ability to reduce ferric ions (Table 5).

#### 2.3.3. Cytotoxic Activity

**Figure 4:**
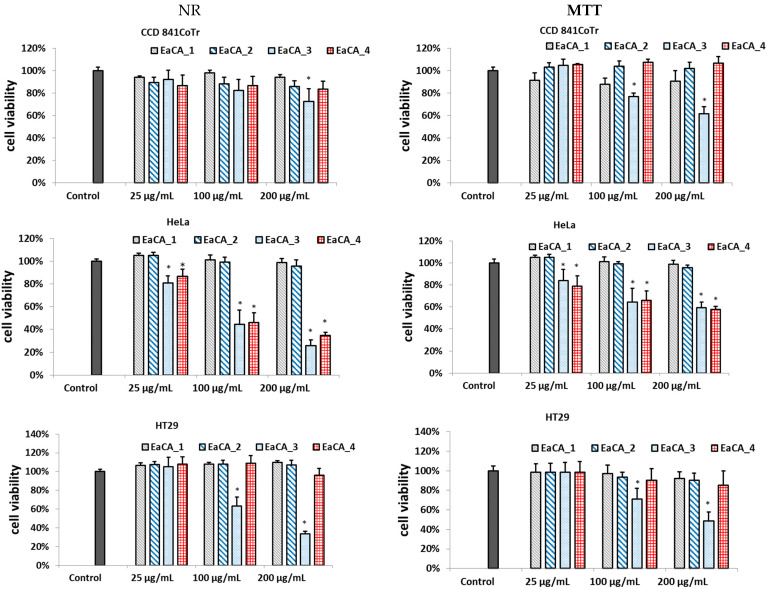
Effect of the different concentrations of subfractions obtained from ethyl acetate extract of *Carlina acaulis* (EaCA) on cell viability determined by the NR and MTT assays expressed as a % of control (0.5% of DMSO in medium). The fractions were obtained using 20% methanol (EaCA_1), followed by 60% methanol (EaCA_2), 80% methanol (EaCA_3), and 100% methanol (EaCA_4). The data are means (*n* = 3) ± SD. One-way ANOVA followed by Dunnett’s post hoc test; the differences were considered significant at *p* < 0.05. * indicates statistically significant difference.

The subfractions were analyzed in terms of cytotoxicity, and the results for the MTT and NR assays are shown in Figure 4.

As can be seen, the subfractions rich in polyphenolic compounds, EaCA_1 and EaCA_2, showed no cytotoxic effect in the tested cell lines. The most active subfraction was EaCA_3, in which fatty acids were detected. It showed the highest activity against HT29 and HeLa cancer lines; however, it also exhibits moderate cytotoxicity against normal epithelial colon cell lines. MTT assay showed that at a concentration of 100 µg/mL, it reduced cell viability to 64%, 71%, and 79% in HeLa, HT29, and CCD 841CoTr, respectively. In the NR test, the values were as follows: 44%, 63%, and 82% in HeLa, HT29, and CCD 841CoTr, respectively.

In turn, EaCA_4 did not negatively affect cell viability and metabolism in CCD 841CoTr. It effectively reduced both parameters in human cervical cancer cells; however, it had no impact on the HT29 line.

## 3. Discussion

*Carlina acaulis* is a plant with great significance in folk medicine; however, it is still poorly known in the context of phytochemical composition. UHPLC-DAD-MS analysis has revealed that the above-ground parts of *C. acaulis* are abundant in phenolic acids as well as flavonoids, mostly belonging to C-glycosides, which seem to be characteristic for the Carlina genus [20]. Some of the detected compounds, namely chlorogenic acid, schaftoside, homoorientin, orientin, vitexin, apigenin, and its 7-glucoside, were previously found in *C. acaulis* [13]; however, it should be noted that the identification was based on a comparison of retention time and UV-VIS spectrum with the standard. The present study not only confirmed these findings using mass data but also showed that *C. acaulis* contains a high amount of other polyphenolic compounds such as dihydroxybenzoic acid, vicenin II, vitexin, and orientin glycosides. Furthermore, it was shown that *C. acaulis* can serve as a rich source of C-glycosides, which are less widespread in nature than O-glycosides. These compounds possess the glycoside moiety attached directly to the flavonoid backbone through C-C covalent bonds, which makes their structure more stable and less susceptible to hydrolysis. This characteristic feature has an impact on their biological properties [21,22]. Some interesting effects have been found for these types of compounds. For example, schaftoside can inhibit the SARS-CoV-2 virus [23], vicenin-II was active against the *Helicobacterium pylori* infection [24] and may prevent osteoarthritis due to inhibition of extracellular matrix degradation [25], and orientin showed neuroprotective, vasodilatation, and cardioprotective activities [26].

Despite the wide application of *C. acaulis* in ethnomedicine, the number of scientific reports on the biological activity of this plant is scarce, and they are mainly focused on carlina oxide, the main volatile component of the essential oil. The present study revealed that *C. acaulis* extract showed cytotoxic potential against human colon cancer cells (HT29) and human cervical cancer cells (HeLa), with a mild cytotoxic effect on normal cell lines. In previous work, the cytotoxicity of this plant was also noted in human malignant melanoma [14].

It was expected that the activity was due to the high polyphenol content because many components identified in the extract showed anticancer potential in in vitro assays. For example, it has been evidenced that chlorogenic acids, which were abundant components of the extract, were effective against human oral squamous carcinoma (HSC-2) and salivary gland tumor (HSG) [27]. In addition, it reduced the proliferation rate, migration/invasion ability, and mitochondrial ATP production in human hepatoma and lung cancer cells [28]. In turn, vitexin and isovitexin decreased the viability of colorectal adenocarcinoma cells [29], and orientin was effective against human bladder carcinoma [30], breast cancer [31], and colorectal carcinoma [32]. Additionally, numerous other flavonoids have demonstrated potential as anticancer agents [33,34,35].

Surprisingly, the activity was not linked to the polyphenol content, as EaCA_1 and EaCA_2 subfractions, which were rich in these components, were not active. On the other hand, subfraction EaCA_3 was highly cytotoxic against both cancer lines and also showed toxicity against normal cells. This may indicate that the presence of polyphenols in the ethyl acetate fraction (EaCA) ameliorated the cytotoxicity of components from EaCA_3 because EaCA was not toxic against normal cells and retained activity toward cancer lines. Such protective activity was previously noted for chlorogenic acid against doxorubicin-induced cytotoxicity in cardiomyocytes [36].

Only three components were identified in the most cytotoxic EaCA_3, including traumatic acid (TA), 9,10-dihydroxy-8-oxooctadec-12-enoic acid, and pinellic acid (PA), which comprise approximately 40.1% of the total yield. Among them, TA may be responsible for the cytotoxic properties of the fraction, as this type of activity of TA was previously reported. TA is an oxidative derivative of unsaturated fatty acids synthesized from linoleic or linolenic acid during plant injury. It plays a role in the protection and repair of plant tissue. Its anticancer activity was described in the literature [37].

Jabłońska-Trypuć et al. have shown that TA is effective against breast cancer cells while simultaneously stimulating proliferation in healthy mammary epithelial cells [38]. It has also been shown that TA exhibits an antioxidant effect in normal human fibroblasts and stimulates ROS in breast cancer cells [39]. Increased ROS production in cells leads to oxidative stress, which, in turn, triggers lipid peroxidation—a detrimental process causing structural modifications to lipid-protein complexes [40]. Literature data have shown that cancer cells are more vulnerable to oxidation compared with normal cells [41,42]. Additionally, it has been found that TA disrupts the GSH/GSSG ratio, an essential antioxidant regulatory system, and induces apoptosis through the caspase pathway [43].

Moreover, there are also some research papers that have documented the cytotoxic properties of pinellic acid against cancer cell lines, specifically HepG2 and PC3 cell lines [44,45].

It should also be noted that the cytotoxic effect of the EaCA_3 subfraction may also be a result of the presence of other components that were not detected by MS and DAD. Therefore, further investigation is needed to clarify this issue. The present study indicates that the anticancer potential of *C. acaulis* is a promising direction for more detailed study. It also justified the traditional use of this plant.

## 4. Materials and Methods

### 4.1. Reagents and Standards

LC-MS-grade methanol, acetonitrile, and formic acid and reference standards were purchased from Merck (Sigma-Aldrich Co., St. Louis, MO, USA). The other solvents (Merck) were analytical grade. Water was purified using Ultrapure Milipore DirectQ 3UV-R (Merck KGaA, Darmstadt, Germany). Reagents, including 2.2-diphenyl-1-picrylhydrazyl (DPPH), o-phenanthroline, and ferric chloride (FRAP reagent), were purchased in Fluka (Sigma-Aldrich Co., St. Louis, MO, USA).

### 4.2. Plant Material

The field cultivation of C. acaulis was conducted following the protocol previously published by Strzemski et al. [46]. Seeds obtained from plants growing in the UMCS Botanical Garden (voucher specimen no. 2005A) were sown in early February in boxes filled with peat substrate. In mid-April, individual seedlings were transplanted into multipallets containing peat substrate. By the beginning of July, the plants had been transferred to loess soil at a density of four plants per square meter. Weeding was performed manually, and no fertilization or protective measures were applied during cultivation. Harvesting took place when the plants began flowering within the first ten days of July in the second year of cultivation.

### 4.3. Extraction and Fractionation

The above-ground parts of the plants were rinsed with running water, dried at room temperature for 48 h, and powdered using a laboratory grinder, IKA A11 (IKA-Werke, Stufen, Germany). Furthermore, the plant material was freeze-dried (0.001 mbar) for 72 h using a Christ Alpha 2-4 LDplus laboratory freeze dryer (Martin Christ Gefriertrocknungsanlagen GmbH, Osterode am Harz, Germany). An amount of 1000 g of dried material was exhaustively extracted with methanol and 70% methanol (3 × 5 L and 3 × 1.5 L for 15 min each) using an ultrasonic bath. The extracts (ECA) were combined, centrifuged at 8000 RPM, filtered, and then concentrated using a vacuum evaporator. They were then frozen and subjected to freeze-drying. The freeze-dried ECA was dissolved in MeOH and subjected to liquid-liquid extractions with n-hexane (5 × 100 mL), followed by ethyl acetate (5 × 100 mL). The resulting residue was evaporated to dryness, suspended in water, and then extracted with n-butanol (5 × 100 mL). The residue obtained after extraction represented the H2OCV fraction. The fractions were concentrated using a vacuum evaporator, frozen, and freeze-dried [20]. Based on the bioactivity assay, the most active fraction (ethyl acetate) was subjected to further fractionation. The ethyl acetate fractions were dissolved in deionized water with the addition of 5% DMSO and then centrifuged (1000× *g*; 10 min). The supernatant was loaded onto a glass preparative column with dimensions of 10 × 30 cm, filled with Cosmosil 75C18—PREP chromatographic packing material (75 μm, Nacalai Tesque), previously impregnated with 1% MeOH in water. Approximately 10 g of the dissolved extract was loaded onto the column at once, and elution was performed using a stepwise gradient of the mobile phase with 3 times the column volume for each concentration. The elution used appropriate amounts of 20%, 60%, and 100% MeOH to obtain the following fractions: EaCA_1 (20% MeOH), EaCA_2 (60% MeOH), EaCA_3 (80% MeOH), and EaCA_4 (100% MeOH).

### 4.4. Chromatographic Analysis (UHPLC—HR/QTOF/MS—CAD–PDA)

The lyophilized samples were dissolved in a mixture of 50% MeOH in water with the addition of 5% dimethyl sulfoxide (DMSO) and then centrifuged and filtered. Chromatographic separation and qualitative analyses were performed using an ultra-high-performance liquid chromatograph (UHPLC) coupled with a charged aerosol detector (CAD) and a high-resolution/quadropole time-of-flight mass spectrometer (HR/QTOF/MS Impact II) equipped with an electrospray ionization (ESI) source. The analyses were carried out on a BEH C18 column (2.1 × 150 mm, 1.7 µm; Waters) at a temperature of 40 °C. The mobile phase consisted of a mixture of acetonitrile with 0.1% formic acid (FA) (solvent B) and water with 0.1% formic acid (FA) (solvent A). A linear gradient elution was performed as follows: from 2% B to 80% B over a period of 30 min, at a flow rate of 0.5 mL/min. MS spectra were acquired in the range of 80–2000 m/z in negative ionization. The working MS parameters were as previously described [20]. The Waters Mass Lynx software was used to process the data. Quantification was carried out using an ultra-performance liquid chromatograph combined with a photodiode detector and a triple quadrupole mass spectrometer (UPLC-PDA-ESI-MS) (ACQUITY TQD, Waters). UV-Vis spectra of the components were registered in the range of 190–750 nm (resolution of 3.6 nm).

### 4.5. Cell Cultures

The HT29 cell line (ATCC^®^ No. HTB-38™) is a human colorectal adenocarcinoma cell line (grade I), derived from a 44-year-old female adult. These cells were cultured in RPMI 1640 medium supplemented with 10% fetal calf serum (FCS) (GibcoTM, Paisley, UK) and antibiotics (100 U/mL penicillin, 100 μg/mL streptomycin, and 0.25 μg/mL amphotericin B) (GibcoTM, Paisley, UK) at 37 °C in a humidified atmosphere with 5% CO_2_.

The CCD 841 CoTr cell line (ATCC^®^ No. CRL-1807™) is a human normal colon epithelial cell line (SV40 transformed), derived from a female at 21 weeks of gestation. These cells were cultured in RPMI 1640 + DMEM (1:1) medium (Sigma-Aldrich Co. LLC, St. Louis, MO, USA) supplemented with 10% FCS and antibiotics at 37 °C in a humidified atmosphere with 5% CO_2_. The human cervical carcinoma cell line (HeLa, ECACC No. 85060701) was also used in this study. Cells were grown as monolayers in 25-cm^3^ culture flasks (Nunc., Roskilde, Denmark) in RPMI 1640 medium supplemented with 5% fetal bovine serum (FBS) (Gibco, Paisley, UK) and antibiotics (100 U/mL penicillin, 100 µg/mL streptomycin) (Gibco, Paisley, UK) at 37 °C in a humidified atmosphere with 5% CO_2_. For cytotoxicity assay, the cells (1 × 10^5^ cells/mL) were plated in 96-well flat-bottom plates, incubated for 24 h at 37 °C, and then treated with the fractions and subfractions for 24 h. Stock solution was prepared using DMSO/culture medium (1:1) and appropriately diluted. The final concentration of DMSO did not exceed 0.5%. Cells treated with 0.5% DMSO in culture medium were taken as controls.

All experiments were performed in triplicate for each extract concentration and presented as a percentage of the control (100%).

### 4.6. Cell Viability Assay

#### 4.6.1. MTT Assay

After incubating the samples for 24 h, a solution of 3-(4,5-dimethylthiazole-2-yl)-2,5-diphenyltetrazolium bromide (MTT) at a concentration of 5 mg/mL (Sigma) was introduced to the cells (25 μL/well), followed by an additional 3-h incubation. The resulting insoluble formazan crystals were dissolved overnight in a mixture containing 10% sodium dodecyl sulfate (SDS) in 0.01 M HCl. The absorbance was gauged at a wavelength of 570 nm using an E-max Microplate Reader (Molecular Devices Corporation, Menlo Park, CA, USA) [47].

#### 4.6.2. Neutral Red Uptake Assay

The procedure was based on Zarandi et al. with minor modifications [48]. Following a 24-h incubation period with the samples, the liquid in the wells was discarded, and the cells were subjected to a 2-h incubation with a neutral red dye solution (40 μg/mL) at 37 °C. Subsequently, the cells were rinsed with phosphate-buffered saline (PBS), and after removing the PBS, decolorizing buffer (150 µL) was introduced. The plates were agitated for 10 min, and the optical density (OD) of the extracted dye was assessed at 540 nm using an E-max Micro-plate Reader (Molecular Devices Corporation, Menlo Park, CA, USA). The outcomes are depicted as the percentage of retained dye quantity in comparison to the control cells (100%).

### 4.7. Antioxidant Activity

#### 4.7.1. DPPH• Free Radical Scavenging Test

The free radical scavenging activity of the tested compounds was evaluated using the 1,1-diphenyl-2-picrylhydrazyl (DPPH•) assay [49]. This method is based on the antioxidant’s ability of to reduce the stable dark violet DPPH• radical (Sigma) to the yellow diphenyl-picrylhydrazine. Briefly, 100 μL of DPPH• solution (0.2 mg/mL in ethanol) was added to 100 μL of various concentrations of the tested compounds (ranging from 0 to 200 μg/mL) as well as to standard Trolox (Sigma) at increasing concentrations (1–50 μg/mL) used as a reference for free radical scavenging activity. After incubating the samples for 20 min at room temperature, the absorbance of the solutions was measured at 515 nm using a microplate reader (BioTek Instruments, Winooski, VT, USA). A lower absorbance value indicates a higher free radical scavenging activity of the compounds. The activity of each substance was determined by comparing its absorbance with that of a blank solution (containing reagents without the compound) and the standard. The capability of each substance to scavenge the DPPH• radical was then calculated using the following formula:

DPPH• scavenging effect (%) = [(X control − X substance/X control) × 100]

where X control is the absorbance of the control and X substance is the absorbance in the presence of tested compound.

#### 4.7.2. Ferric-Reducing Antioxidant Power Assay (FRAP)

Each tested compound concentration was dissolved in Milli-Q water and then mixed with an equal volume of 0.2 M sodium phosphate buffer (pH 6.6) and 1% potassium ferricyanide. The mixture was incubated for 30 min at 37 °C. After the incubation, 10% trichloroacetic acid (*w*/*v*) was added to the mixture, and the resulting solution was centrifuged at 1000× *g* for 5 min. Next, 1 mL of the upper layer was mixed with an equal volume of Milli-Q water and 0.1% ferric chloride. The absorbance of the solution was measured at 700 nm using a spectrophotometer (BioTek Instruments, Winooski, VT, USA). Ascorbic acid was used as the positive control in this assay [20].

### 4.8. Statistical Analysis

All analyses were conducted three times. The obtained data were subjected to statistical analysis using Statistic version 13.3 software. One-way ANOVA was performed, followed by Dunnett’s post hoc test. The results were presented as the mean ± standard deviation (SD). Statistical significance was determined at a *p*-value of ≤ 0.05.

## 5. Conclusions

In the present work, the phytochemical composition and cytotoxic potential of *C. acaulis* extract against human colorectal adenocarcinoma (HT29) and human cervical cancer (HeLa) cells were investigated. The investigation revealed that *C. acaulis* is a rich source of phenolic acids and flavonoids, with predominant chlorogenic acids and C-glycosides of luteolin and apigenin. Among the flavonoids, kaempferol dihexosidipentose and schaftoside were the most abundant, followed by isoorientin, vitexin-2-O-rhamnoside, and vicenin II. Furthermore, it has been demonstrated that *C. acaulis* shows significant cytotoxicity against the tested cell lines. However, the effect was not linked to the presence of polyphenolic compounds. The subfraction containing oxylipins belonging to oxygenated fatty acids was the most active and decreased the viability of HeLa and HT29 cells with minimal negative effects on normal cells.

The present study indicates that the anticancer potential of *C. acaulis* is a promising direction for more detailed study. In the future, the work should be focused on exploring the molecular mechanisms connected with the biological activity of the plant. Furthermore, the isolation of specific components should be carried out to identify the component with a specific biological effect.

## Figures and Tables

**Table 1:** Compounds found in the extract of aerial parts of *C. acaulis*. The quantification is expressed per gram of dried material.

Nr	RT (min)	M/Z	MS2	Ion Formula [M/Z-H]	Δppm	Identified	Amount (mg/g DW)
1	0.8	191.055878	191, 135	C_7_H_11_O_6_	1.2	quinic acid	0.99 ± 0.01
2	1.7	153.019025	153, 109	C_7_H_5_O_4_	2.0	dihydroxybenzoic acid	2.14 ± 0.01
3	2.3	343.102756	343, 135	C_15_H_19_O_9_	2.0	unknown	
4	2.5	353.087070	353, 351, 191, 133	C_16_H_17_O_9_	2.1	3-caffeoylquinic acid	3.49 ± 0.03
5	2.6	353.087049	353, 191	C_16_H_17_O_9_	2.1	5-caffeoylquinic acid	9.21 ± 0.01
6	3.0	373.113574	373, 165, 150	C_16_H_21_O_10_	1.2	unknown	
7	3.3	609.145133	609, 489, 399, 369	C_27_H_29_O_16_	1.6	luteolin di-C-glucoside	0.69 ± 0.01
8	3.5	337.092161	337, 191	C_16_H_17_O_8_	2.2	5-p-coumaroylquinic acid	0.57 ± 0.06
9	3.9	593.150084	593, 473, 383, 353	C_27_H_29_O_15_	1.9	vicenin II	1.92 ± 0.01
10	4.3	609.145243	609, 489, 429, 357	C_27_H_29_O_16_	1.4	2”-isoorientin O-glucopyranoside	0.41 ± 0.01
11	4.4	563.139696	563, 473, 443, 383, 353	C_26_H_27_O_14_	1.7	shaftoside	3.12 ± 0.01
12	4.6	447.092277	447, 429, 357, 327, 297	C_21_H_19_O_11_	2.3	orientin	1.05 ± 0.01
13	4.8	563.139620	563, 503, 473, 443, 383, 353	C_26_H_27_O_14_	1.8	isoshaftoside (I)	1.06 ± 0.01
14	4.9	579.134349	579, 459, 429, 357, 327, 309	C_26_H_27_O_15_	2.1	isoorientin	1.84 ± 0.01
15	5.2	563.139889	563, 473, 443, 383, 353	C_26_H_27_O_14_	1.3	isoshaftoside (II)	1.22 ± 0.01
16	5.3	593.150261	593, 473, 383, 293	C_27_H_29_O_15_	1.6	isovicenin	1.00 ± 0.01
17	5.5	417.081764	417, 357, 327, 297	C_20_H_17_O_10_	2.3	6-C-xylosyl luteolin	0.97 ± 0.01
18	5.6	577.155385	577, 457, 413, 341, 311, 293	C_27_H_29_O_14_	1.5	vitexin-2-O-rhamnoside	1.96 ± 0.01
19	5.8	525.233345	525, 481, 319, 301, 119	C_25_H_34_O_12_	1.5	lucidumoside A	
20	6.3	577.155674	577, 457, 445, 427, 324	C_27_H_29_O_14_	1.0	isovitexin 2″-O-rhamnoside	1.74 ± 0.01
21	6.4	435.412847	435, 273, 167	C_21_H_24_O_10_	1.7	phlorizin	
22	6.5	413.413442	413, 269, 161	C_19_H_25_O_10_	2.1	unknown	
23	9.2	771.343701	771,609, 489, 447, 343, 301	C_37_H_55_O_17_	1.0	hesperitin 7-O diglucosorhamnoside	0.20 ± 0.01
24	9.7	741.168209	741, 285	C_32_H_38_O_20_	1.3	kaempferol dihexosodipentose	3.14 ± 0.01
25	11.2	643.354506	643, 625, 481, 319, 113	C_27_H_33_O_18_	0.2	gossypetin dihexose	0.57 ± 0.06
26	12.7	227.128536	227, 183, 165	C_12_H_19_O_4_	1.5	traumatic acid	2.39 ± 0.01
27	13.0	327.217412	327, 211, 171	C_18_H_31_O_5_	0.9	9,10-dihydroxy-8-oxooctadec-12-enoic acid	1.64 ± 0.02
28	14.2	329.232438	329, 229, 211, 171	C_18_H_33_O_5_	2.8	pinellic acid	2.06 ± 0.02
29	16.6	311.185991	311, 293, 267	C_18_H_32_O_2_	1.3	octadecadienoic acid derivative	
30	21.8	562.313912	562, 502, 277, 224	C_27_H_49_NO_9_P	2.0	phospholipids	
31	22.2	505.255694	505, 277, 152	C_21_H_34_N_10_O_3_P	0.3	phospholipids	
32	22.6	595.288490	595, 279, 241, 152	C_24_H_40_N_10_O_6_P	−1.6	phospholipids	
33	23.1	564.329193	564, 504, 279, 224	C_30_H_42_N_7_O_4_	2.1	phospholipids	

Compounds were identified based on Compound Crawler Bruker, Sirius 4.0.1. and confirmed by standards when available.

**Table 2:** Quantitative analysis of the fractions, expressed as mg per gram of dried extract (±SD).

No.	Compounds	HCA	EACA	BCA	H_2_OCA
1	quinic acid	ND	ND	ND	1.01 ± 0.01
2	dihydroxybenzoic acid	ND	ND	25.05 ± 0.07	0.01 ± 0.00
3	ND	ND	ND	ND	+
4	3-caffeoylquinic acid	ND	65.41 ± 0.08	17.37 ± 0.38	0.02 ± 0.00
5	5-caffeoylquinic acid	ND	172.85 ± 0.11	25.87 ± 0.07	0.06 ± 0.01
6	ND	ND	ND	+	ND
7	luteolin di-C-glucoside	ND	12.30 ± 0.14	0.91 ± 0.06	ND
8	5-p-coumaroylquinic acid	ND	10.63 ± 1.08	3.00 ± 0.07	0.02 ± 0.00
9	vicenin II	ND	34.17 ± 0.08	1.42 ± 0.01	ND
10	2”-isoorientin O-glucopyranoside	ND	ND	2.85 ± 0.08	ND
11	shaftoside	ND	55.56 ± 0.08	2.32 ± 0.01	ND
12	orientin	ND	ND	7.75 ± 0,10	ND
13	isoshaftoside (I)	ND	18.90 ± 0.14	0.79 ± 0.01	ND
14	isoorientin	ND	ND	15.06 ± 0,12	ND
15	isoshaftoside (II)	ND	21.69 ± 0.08	0.90 ± 0.01	ND
16	isovicenin	ND	17.89 ± 0.17	0.75 ± 0.01	ND
17	6-C-xylosyl luteolin	ND	17.35 ± 0.08	0.72 ± 0.02	ND
18	vitexin-2-O-rhamnoside	ND	35.00 ± 0.08	1.46 ± 0.02	ND
19	lucymidozyd A	ND	+	+	ND
20	isovitexin 2″-O-rhamnoside	ND	31.08 ± 0.08	1.30 ± 0.03	ND
21	phlorizin	ND	ND	+	ND
22	ND	ND	+	+	ND
23	hesperitin 7-O diglucosorhamnoside	ND	2.92 ± 0.08	ND	ND
24	kaempferol dihexosodipentose	ND	45.19 ± 0.08	ND	ND
25	gossypetin dihexose	ND	8.15 ± 0.83	ND	ND
26	traumatic acid	ND	33.7 ± 0.01	ND	ND
27	9,10-dihydroxy-8-oxooctadec-12-enoic acid	ND	10.81 ± 0.03	ND	ND
28	pinellic acid	ND	11.31 ± 0.01	ND	ND
29	octadecadienoic acid derivative	ND	+	ND	ND
30	phospholipids	+	ND	ND	ND
31	phospholipids	+	ND	ND	ND
32	phospholipids	+	ND	ND	ND
33	phospholipids	+	ND	ND	ND

ND—not detected.

**Table 3:** The results (±SD) of radical scavenging activity (DPPH) and ferric reducing antioxidant power (FRAP) obtained for hexane (HCA), ethyl acetate (EaCA), butanol (BCA), and water (H_2_OCA) fractions from the methanolic extract of *C. acaulis*.

Fractions	Concentration (µg/mL)	Equivalent of Trolox Concentration	Equivalent of Ascorbic Acid Concentration
H_2_OCA	25	6.419 ± 0.211	4.997 ± 0.347
	100	18.147 ± 0.166	26.362 ± 0.638
	200	31.915 ± 0.338	51.978 ± 0.549
BCA	25	8.151 ± 0.463	4.253 ± 0.213
	100	16.809 ± 0.291	22.110 ± 0.204
	200	30.077 ± 0.191	46.451 ± 0.814
EaCA	25	10.456 ± 0.264	8.399 ± 0.347
	100	22.344 ± 0.337	38.585 ± 1.994
	200	38.480 ± 0.544	70.685 ±1.125
HCA	25	3.572 ± 1.255	0.214 ± 0.213
	100	5.814 ± 0.203	4.678 ± 0.233
	200	8.856 ± 0.771	11.587 ± 0.245

Equivalent of ascorbic acid/Trolox—the reducing/antioxidant power of the extract at a given concentration is equivalent to the reducing power of a given concentration of ascorbic acid/Trolox.

**Table 4:** Quantitative analysis of the subfractions obtained using 20% methanol (EaCA_1), followed by 60% methanol (EaCA_2), 80% methanol (EaCA_3), and 100% methanol (EaCA_4) expressed as mg per gram of dried extract (±SD).

No.	Compound	Amount (mg/g d.w. of Fraction)			
		EaCA_1	EaCA_2	EaCA_3	EaCA_4
1	3-caffeoquinic acid	113.77 ± 1.05	ND	ND	ND
2	5-caffeoquinic acid	300.627 ± 0.19	ND	ND	ND
3	5-p-coumarylquinic acid	18.49 ± 1.88	ND	ND	ND
4	luteolin di-C-glucoside	ND	26.10 ± 0.38	ND	ND
5	vicenin	ND	72.50 ± 0.22	ND	ND
6	2”-isoorientin O-glucopyranoside	ND	15.38 ± 0.22	ND	ND
7	schaftoside	ND	117.89 ± 0.23	ND	ND
8	isoschaftoside I	ND	40.10 ± 0.38	ND	ND
9	isoschaftoside II	ND	46.02 ± 0.22	ND	ND
10	isovicenin	ND	37.95 ± 0.44	ND	ND
11	6-C-xylosyl luteolin	ND	36.82 ± 0.21	ND	ND
12	vitexin 2″-O-rhamnoside	ND	74.27 ± 0.24	ND	ND
13	isovitexin 2″-O-rhamnoside	ND	65.94 ± 0.23	ND	ND
14	hesperitin 7-O diglucosorhamnoside	ND	7.69 ± 0.22	ND	ND
15	dihexosodipentose kaempferol	ND	118.90 ± 0.14	ND	ND
16	dihexose gossypetin	ND	21.44 ± 2.18	ND	ND
17	traumatic acid	ND	ND	143.08 ± 0.11	ND
18	9,10-dihydroxy-8-oxsooctadec-12-enic acid	ND	ND	129.56 ± 0.04	ND
19	pinellic acid	ND	ND	136.85 ± 0.24	ND

ND—not detected.

**Table 5:** The results (±SD) of radical scavenging activity (DPPH) and ferric reducing antioxidant power (FRAP) for subfractions obtained from ethyl acetate (EaCA) fractions of the extract from *C. acaulis*.

Fractions	Concentration (µg/mL)	Equivalent of Trolox Concentration	Equivalent of Ascorbic Acid Concentration
EaCA_4	25	0.891 ± 0.200	0
	100	1.003 ± 0.587	0
	200	1.079 ± 0.226	1.778 ± 0.789
EaCA_3	25	1.851 ± 0.202	0
	100	4.145 ± 0.264	4.509 ± 0.263
	200	6.493 ± 0.249	11.679 ± 0.436
EaCA_2	25	12.156 ± 1.499	14.670 ± 0.407
	100	28.983 ± 0.166	53.785 ± 1.063
	200	41.742 ± 0.041	99.490± 2.629
EaCA_1	25	12.081 ± 0.274	19.134 ± 0.213
	100	32.882 ± 0.249	79.295 ± 0.943
	200	42.953 ± 0.106	152.955 ± 4.209

Equivalent of ascorbic acid/Trolox—the reducing/antioxidant power of the extract at a given concentration is equivalent to the reducing power of a given concentration of ascorbic acid/Trolox.

## Data Availability

Data are contained within the article or Supplementary Materials.

## Supplementary Materials

The following supporting information can be downloaded at: https://www.mdpi.com/article/10.3390/molecules28166148/s1. Figure S1: Chromatograms obtained from ultra-performance liquid chromatography with mass spectrometry and electrospray ionization UHPLC-ESI-MS(-) (**a**) and UHPLC-ESI-MS (+) (**b**) and UHPLC with photodiode detector—PDA (254 nm) (**c**). These chromatograms represent fractions obtained through liquid–liquid extraction from the extract of *C. acaulis*.
