# Supplementary material for: Polyphenolic Composition of Carlina acaulis L. Extract and Cytotoxic Potential against Colorectal Adenocarcinoma and Cervical Cancer Cells
Source: Molecules. 2023 Aug 20;28(16):6148. doi: 10.3390/molecules28166148 (PMC10458490; doi:10.3390/molecules28166148)
Supplement: Supplementary file 1 [file molecules-28-06148-s001.zip › molecules-2556745-supplementary.pdf]

Polyphenolic composition of *Carlina acaulis* L. extract and cytotoxic potential against colorectal adenocarcinoma and cervical cancer cells

Ireneusz Sowa[1,*], Jarosław Mołdoch[2], Roman Paduch[3], Maciej Strzemski[1], Jacek Szkutnik[4], Katarzyna Tyszczuk-Rotko[5], Sławomir Dresler[1], Dariusz Szczepanek[6], and Magdalena Wójciak[1,*]

[1] Department of Analytical Chemistry, Medical University of Lublin, Chodźki 4a, 20-093 Lublin, Poland; i.sowa@umlub.pl (I.S.); maciej.strzemski@poczta.onet.pl (M.S.); dresler.slawomir@gmail.com (S.D.); magdalena.wojciak@umlub.pl (M.W.);

[2] Department of Biochemistry and Crop Quality, Institute of Soil Science and Plant Cultivation, State Research Institute, 24-100 Puławy, Poland; jmoldoch@iung.pulawy.pl (J.M.)

[3] Department of Virology and Immunology, Institute of Biological Sciences, Faculty of Biology and Biotechnology, Maria Curie-Skłodowska University, 19 Akademicka Street, 20-033 Lublin, Poland, roman.paduch@mail.umcs.pl (R.P.)

[4] Independent Unit of Functional Masticatory Disorders, Medical University of Lublin, 20-093 Lublin, Poland; jacek.szkutnik@umlub.pl

[5] Institute of Chemical Sciences, Faculty of Chemistry, Maria Curie-Skłodowska University in Lublin, 20-031 Lublin, Poland; katarzyna.tyszczuk-rotko@mail.umcs.pl

[6] Chair and Department of Neurosurgery and Paediatric Neurosurgery, Medical University of Lublin, 20-090 Lublin, Poland, dariusz.szczepanek@umlub.pl (D.S.)

* Correspondence: ireneusz.sowa@umlub.pl (I.S.); magdalena.wojciak@umlub.pl (M.W.)

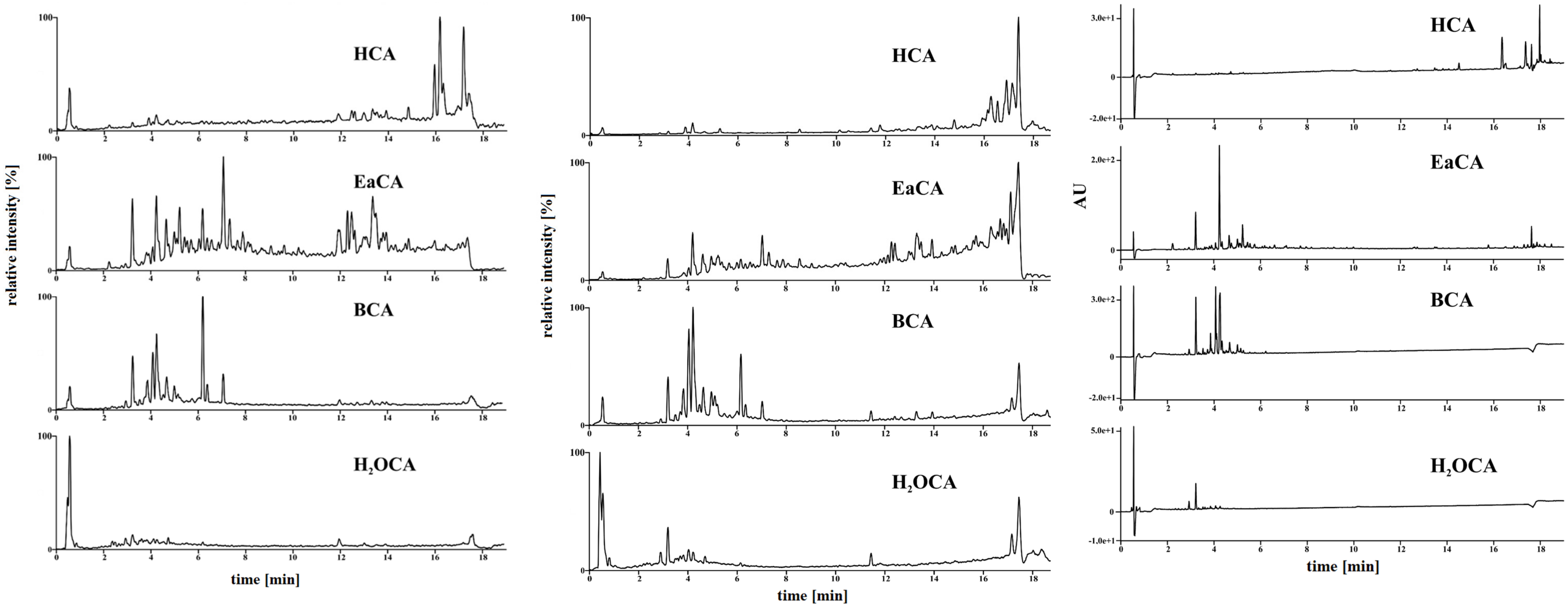

(a) (b) (c)

Figure S1. Chromatograms obtained from ultra-performance liquid chromatography with mass spectrometry and electrospray ionization UHPLC-ESI-MS(-) (a) and UHPLC-ESI-MS(+) (b), and UHPLC with photodiode detector—PDA (254 nm) (c). These chromatograms represent fractions obtained through liquid–liquid extraction from the extract of *C. acaulis*. The abbreviations used are as follows: ECV—methanol extract, HCV—hexane fraction, EaCV—acetate fraction, BCV—butanol fraction, H_2OCV—water fraction.
